# Linking extreme interannual changes in prey availability to foraging behaviour and breeding investment in a marine predator, the macaroni penguin

**DOI:** 10.1371/journal.pone.0184114

**Published:** 2017-09-14

**Authors:** Cat Horswill, Philip N. Trathan, Norman Ratcliffe

**Affiliations:** 1 British Antarctic Survey, Natural Environment Research Council, High Cross, Cambridge, United Kingdom; 2 Institute of Biodiversity, Animal Health & Comparative Medicine, University of Glasgow, Glasgow, United Kingdom; Centre National de la Recherche Scientifique, FRANCE

## Abstract

Understanding the mechanisms that link prey availability to predator behaviour and population change is central to projecting how a species may respond to future environmental pressures. We documented the behavioural responses and breeding investment of macaroni penguins *Eudyptes chrysolophus* across five breeding seasons where local prey density changed by five-fold; from very low to highly abundant. When prey availability was low, foraging trips were significantly longer and extended overnight. Birds also foraged farther from the colony, potentially in order to reach more distant foraging grounds and allow for increased search times. These extended foraging trips were also linked to a marked decrease in fledgling weights, most likely associated with reduced rates of provisioning. Furthermore, by comparing our results with previous work on this population, it appears that lowered first-year survival rates associated, at least partially, with fledging masses were also evident for this cohort. This study integrates a unique set of prey density, predator behaviour and predator breeding investment data to highlight a possible behavioural mechanism linking perturbations in prey availability to population demography.

## Introduction

An increasing number of studies have associated the population dynamics of wild species with climate and anthropogenic pressures [1,2]. Understanding the primary causes and underlying mechanisms that determine these relationships is crucial for projecting long-term population changes. As yet, few studies have been able to successfully isolate these mechanisms (but see [3,4]). This is because long-term studies on the dynamics of food webs are largely lacking due to the associated logistic and financial commitments.

In marine ecosystems, adverse conditions need to be integrated over relatively short food chains [5]. For central-place foragers, such as seabirds and seals, this constraint can be further intensified by restricted foraging ranges. For example, in the Southern Ocean, large breeding aggregations of seabirds and seals depend on local swarms of Antarctic krill *Euphausia superba* as a key food resource during the breeding season [6–9]. Therefore, fluctuations in krill density can substantially impact the diet, activity budgets, and levels of breeding investment employed by these populations [6].

Macaroni penguins *Eudyptes chrysolophus* are one of the most important avian marine predators in the sub-Antarctic region, reported to consume more prey than any other seabird species within this ecosystem [10]. During winter, macaroni penguins are fully pelagic [11], however in early chick-rearing, or brood-guard, their foraging range is constrained by a requirement to provision the chick at the colony on a daily basis. Chick provisioning during brood-guard is conducted exclusively by the female, whilst the male broods and guards the chick on-shore [12], and foraging trips typically follow a diurnal pattern; leaving the colony at dawn and returning before dusk [13–21]. Dietary studies indicate that changes in target prey species and primary productivity often occur concurrently with reduced rates of provisioning, chick growth [18] and fledging mass [6], as well as altered foraging ranges in macaroni penguins [22]. However, as yet, very few studies on marine predators include estimates of prey abundance collected independently of predator diet (but see [6,23]).

This study combines acoustic estimates of local krill abundance with behavioural data collected from macaroni penguins during early chick-rearing, i.e. when central-place constraints are greatest. Data were collected across five consecutive breeding seasons where a five-fold change in local krill density occurred; from very low to highly abundant conditions (24 g m^-2^ to 137 g m^-2^; [24]). Concurrent changes in fledging mass are also assessed in order to highlight a possible behavioural mechanism that links fluctuations in prey abundance to population demography.

## Materials and methods

### Species and study site

The foraging behaviour of female macaroni penguins breeding at the Fairy Point colony, Bird Island, South Georgia (54° 00’ S, 38° 03’W), was monitored during the brood-guard phase of five consecutive breeding seasons; 2001 to 2005. On Bird Island, an individual breeding season lasts from egg-laying in November to chick-fledging in the subsequent March. From here on, a breeding season is referred to by the year that the chick fledged. The number of breeding pairs at Fairy Point was stable during the study period (mean = 505, SD = 33). Annual estimates of population fledging mass were collected shortly before fledging (16–19 February) by weighing 100 to 106 chicks to the nearest 0.05 kg with a Pesola spring balance. The sample size of chicks weighed was proportionally large compared to the total number fledging (~35% of chicks fledging) and therefore considered to be representative of the whole colony.

**Fig 1:**
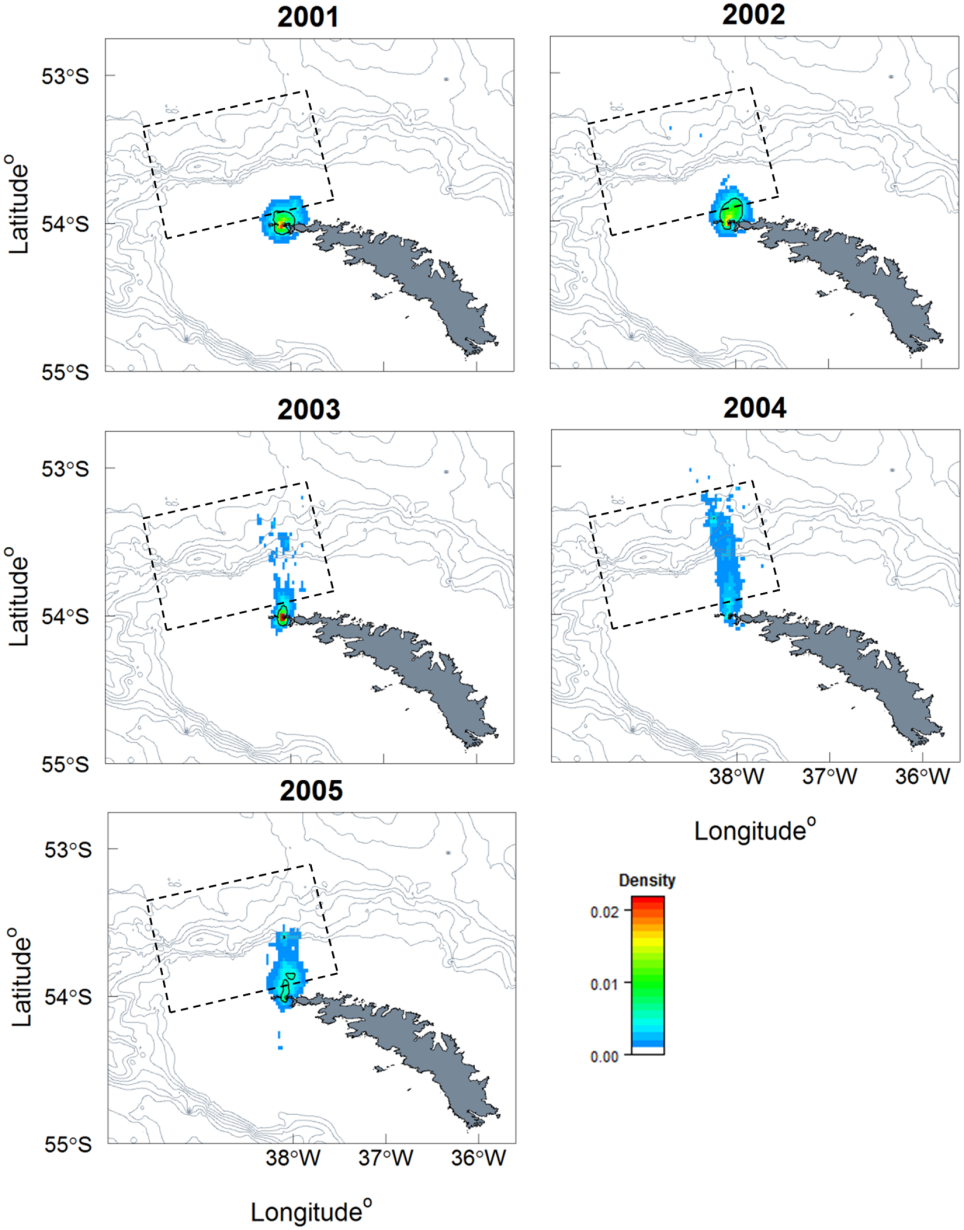
Density and distribution of foraging trips conducted by female macaroni penguins during brood-guard, 2001–2005. Maps show South Georgia and 500m bathymetric contour lines. The edge of the continental shelf was taken at 500m (projection: South Georgia Lambert Conformal Conic). Density contour shown for 0.5% in black solid line, the acoustic survey area for krill is shown as a dashed box.

Acoustic transect surveys were carried out annually during the chick-rearing period (December-February). The survey area was a standardised box (133 km by 80 km) to the northwest of South Georgia that spanned the continental shelf break and overlapped the foraging range of macaroni penguins during brood-guard (Fig 1). Each survey consisted of eight 80 km parallel transects [24]. Local krill density changed by five-fold during the five study years (Table 1; [24]). The density of krill was very low during 2001 and 2004. This was also coupled with a low coefficient of variation (CV) during 2004, indicating either a small number of krill sparsely distributed or a small number of krill regularly distributed with low spatial variability [24]. In contrast, the density of krill was abundant in 2003 and 2005, and highly abundant in 2002 (Table 1; [24]). In 2003 and 2005 high density of krill occurred alongside high CV values, indicating high spatial variability; i.e. irregular distribution of dense swarms. In contrast, krill density in 2002 appeared to be high with low spatial variability; i.e. krill was consistently abundant across the area.

**Table 1:** Annual sample size for tracking of macaroni penguins at Bird Island, South Georgia, with local krill densities (coefficient of variance), 2001–2005.

Year	TDR		ARGOS		Krill density (CV)
	No. birds	Deployment duration (days ±sd)	No. birds	Deployment duration (days ±sd)	
2001	7	3.71 ±0.25	8	2.41 ±0.73	37 (27)
2002	10	2.83 ±0.58	20	2.05 ±1.08	137 (30)
2003	9	5.13 ±1.58	11	4.93 ±0.81	85 (58)
2004	11	6.42 ±2.54	11	4.81 ±2.20	26 (10)
2005	8	5.54 ±1.58	20	3.65 ±1.27	89 (61)

### Foraging data

Dive profiles and spatial distribution were monitored using time-depth recorders (TDR) and ARGOS satellite-transmitters (PTT), respectively (see Table 1 for sample sizes and mean deployment durations). Only one type of device was deployed on each individual because the combined mass was too great for tandem deployment without risking adverse effects on bird behaviour [25]. The TDRs (Wildlife Computers Mk 7, [95 ×15 ×15 mm, 50g], depth resolution ±1m) were less than 1.0% of the adult body mass, and were salt water activated to record time and depth every 2 seconds. The PTTs (Telonics ST10 devices, Mesa [95 ×42 ×20 mm, 85 g], Sirtrack Kiwisat 10, Havelock North [130 ×35 ×20 mm, 100 g] and Wildlife Computers SPOT4, Redmond [90 ×20 ×15 mm, 70g]) (Table 1) were <2.25% of the adult mass. All devices were attached dorsally using waterproof tape (Tesa AG, Hamburg), quick-setting 2-part epoxy glue (RS components, Corby) and cable ties. Because the devices were deployed as part of independent studies the uplink frequencies varied across the years but were typically at an interval of 60 seconds. Fieldwork was approved by the British Antarctic Survey Animal Ethics Committee.

### Metrics of foraging behaviour

Metrics of diving behaviour were estimated using Matlab^™^ code developed by [26]. The sea surface for each TDR record was calibrated to remove fluctuations and allow resolution of the water surface (zero offset correction). To minimise bias associated with the resolution of the TDRs, any dives shallower than 3m in depth were discarded. Because this study was primarily focused on analysing foraging behaviour, travelling (non-foraging) dives were removed. Travelling dives were classified as shallower than 15m and lasting less than 45 seconds [27] (verified by bimodal histogram plots). This classification was not applied to nocturnal foraging activity because nocturnal dives predominantly occurred at depths shallower than 20m [12,13,19]. The distinction between local day and night was defined using the median value for nautical twilight for each deployment, calculated using the global location, date and GMT offset (http://www.sunrisesunset.com/custom_srss_calendar2.asp; accessed 10 June 2011; S1 Table). Mean maximum dive depth and mean vertical distance travelled during the bottom phase of a dive (the vertical undulations near the bottom of the dive when the bird is thought to be actively foraging, e.g. [28]) were determined for each dive.

The maximum distance travelled from the colony (km) and the most likely path that a penguin followed were derived from the PTT data (R packages *trip* and *tripEstimation*; [29,30]). Intermediate points were estimated with uncertainty in a Bayesian Markov chain Monte Carlo model, where a log-normal distribution was used to stochastically assign initial swim speeds (mean = 2 km hr^-1^, SD = 1; derived from satellite-tracking of rockhopper penguins *Eudyptes chrysocome*; [31]). Observation error was based on the respective error of the ARGOS Location Class (LCB 80 km; LCA 50 km; LC0 20 km; LC1 10 km; LC2 8 km; LC3 4 km). Five chains of 2000 iterations were simulated, and the initial 500 iterations were discarded as burn-in. The most likely path that a penguin followed was derived from the posterior estimate of the time-series of primary locations. Repeated trips by individuals were separated using a spatial buffer around the colony that was equal to the modal error class of the PTT data (LQ1; 10 km). Any trips that contained a single location fix were removed. The duration of each foraging trip was calculated as the time interval between an individual leaving and returning to the buffer zone, and the maximum distance from the colony per trip was used as a proxy for foraging range.

To examine interannual variation in foraging dispersal, a map of time spent was constructed across a 0.2° resolution grid. The time spent within each grid cell was summed for all of the tracked birds within each year based on the primary location fixes and the possible intermediate locations that a penguin may have visited [32]. This was divided by the total at-sea time for that year to estimate the proportion of time spent across the area and to examine foraging dispersal (or aggregation).

### Statistical analysis

In agreement with [26], the analyses of dive depth and vertical distance travelled whilst foraging used deployment means. Here, interannual differences were assessed using generalised least squares and a variance weighting for year. Mixed effects models were used to examine annual variation in trip duration and maximum distance, as well as the relationship between these metrics of foraging behaviour. Each model included year as a fixed factor to account for changes in environmental conditions. The mixed-effect models also included a model-specific random effect structure (R package *nlme*, [33]). These were: (1) a generalised least squares model with no random effect; (2) a mixed effects model with a random intercept for each individual; and (3) a mixed effects model with a random intercept and slope for each individual. Models with different random effects structures were compared using restricted maximum likelihood (REML) estimation and the Akaike information criterion (AIC) [34,35]. A difference (ΔAIC) of less than 2 AIC units was taken to suggest that competing models received a similar amount of support from the data. The best candidate model was also re-fitted using a constant variance function for year. Normality of residuals was checked using the Shapiro-Wilk test (R package *car* [36]).

### The effect of foraging behaviour and fledging mass

The relationship between foraging behaviour and fledging mass was examined using linear regression. The available degrees of freedom prevented the inclusion of all foraging metrics and therefore annual means for dive depth and trip duration were included to represent different aspects of the activity budget. Interannual variation in fledging mass was examined using an ANOVA and post-hoc Tukey test.

## Results

### Interannual variation in foraging parameters

**Fig 2:**
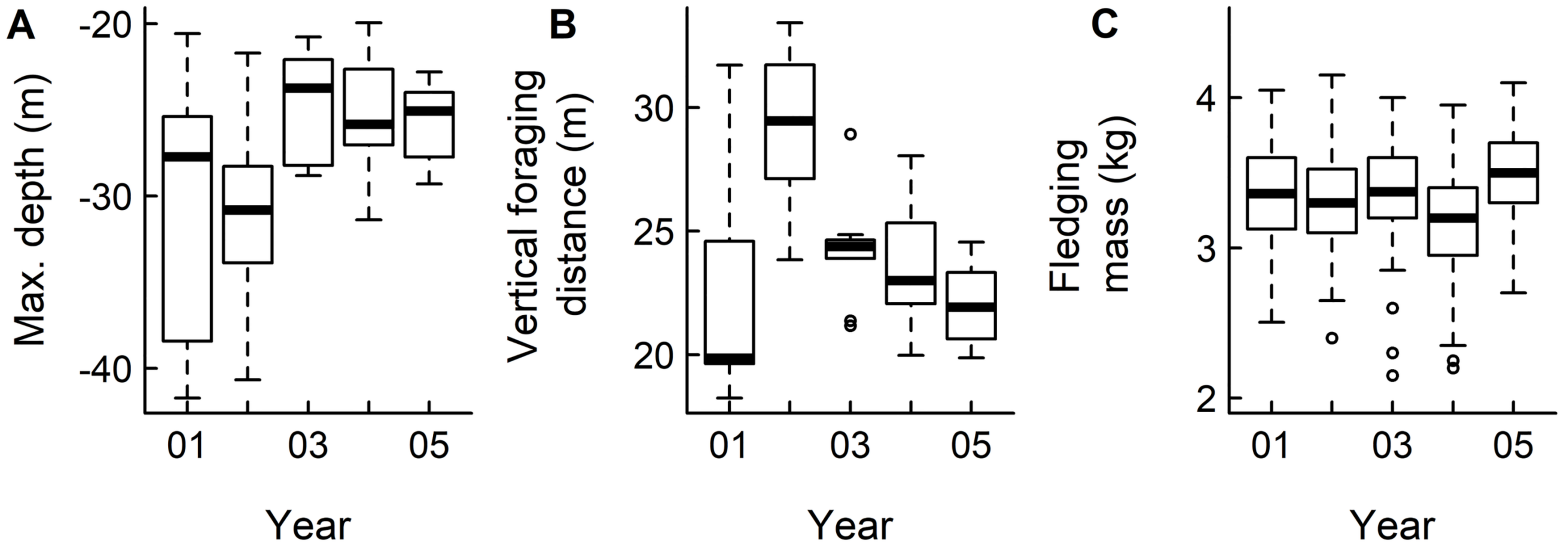
Annual diving behaviour of macaroni penguins during brood-guard and annual fledging masses, 2001–2005 (boxes show mean ± interquartile range, whiskers indicate the reasonable extremes of the data and points are outliers). A) mean maximum depth (m) of foraging dives per deployment; B) mean vertical distance travelled during the bottom phase of a dive (m) per deployment; C) population fledging masses (kg).

The TDR dataset contained 51,861 individual foraging dive profiles from 45 female macaroni penguins. Diving depths did not significantly change between years (GLS: all values p > 0.05), however, variability in diving depth increased during 2001 (reference *σ*^*2*^ = 1.0) and 2002 (ratio to reference *σ*^*2*^ = 0.66), compared to 2003 (ratio to reference *σ*^*2*^ = 0.40), 2004 (ratio to reference *σ*^*2*^ = 0.41) and 2005 (ratio to reference *σ*^*2*^ = 0.28) (Fig 2A). In addition, vertical distance travelled during the bottom phase of a dive significantly increased during 2002 (GLS: t = 2.89, p < 0.01), and variation between individuals was greater in 2001 (reference *σ*^*2*^ = 1.0) and 2002 (ratio to reference *σ*^*2*^ = 0.63), compared to 2003 (ratio to reference *σ*^*2*^ = 0.43), 2004 (ratio to reference *σ*^*2*^ = 0.54) and 2005 (ratio to reference *σ*^*2*^ = 0.32) (Fig 2B).

**Fig 3:**
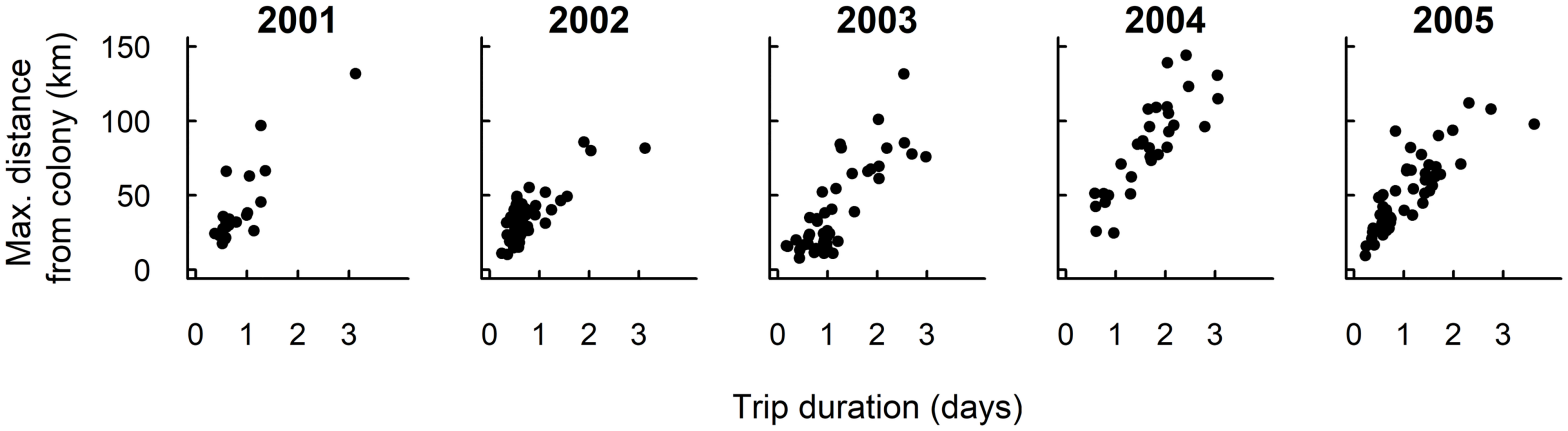
The relationship between maximum distance travelled from the colony and trip duration for female macaroni penguins during brood-guard, 2001–2005.

The PTT dataset contained 226 individual foraging trips from 70 female macaroni penguins. The original dataset contained 4781 fixes and the imputed dataset contained 4854 positions. The data were best described by a model that included a random intercept term for each individual, with a large variance between individuals (GLMM: *d*^2^ = 21.47, *df* = 8, ΔAIC = 0). The individual term accounted for 10% of the residual variance. The model without a random effect (GLMM: *df* = 7, ΔAIC = 6.41), and the model that included a random intercept and slope term for each individual (GLMM: *df* = 28, ΔAIC = 9.43) were not retained. Maximum distance travelled from the colony also significantly increased with trip duration (GLMM: t = 20.97, p<0.001), and the average trip duration and maximum distance from the colony changed between years (Figs 1 and 3). The number of short distance trips with a longer duration increased during 2003 (GLMM: t = -2.31, p = 0.02), whilst trips were predominantly longer than 1 day with greater maximum distances during 2004 (GLMM: t = 3.14, p<0.01) (Figs 1 and 3). In addition, variation in these metrics of foraging behaviour was greatest in 2004 (ratio to reference *σ*^*2*^ = 1.09) and 2003 (ratio to reference *σ*^*2*^ = 1.06), lowest in 2002 (ratio to reference *σ*^*2*^ = 0.60) and 2005 (ratio to reference *σ*^*2*^ = 0.84), with 2001 as the reference variance (*σ*^*2*^ = 1.0). Finally, foraging dispersal was lowest during 2001 and 2002, and highest in 2004 (Fig 1). It was not possible to demark an area with a foraging density greater than 0.5% during 2004 (Fig 1).

### The effect of foraging behaviour on fledging mass

**Fig 4:**
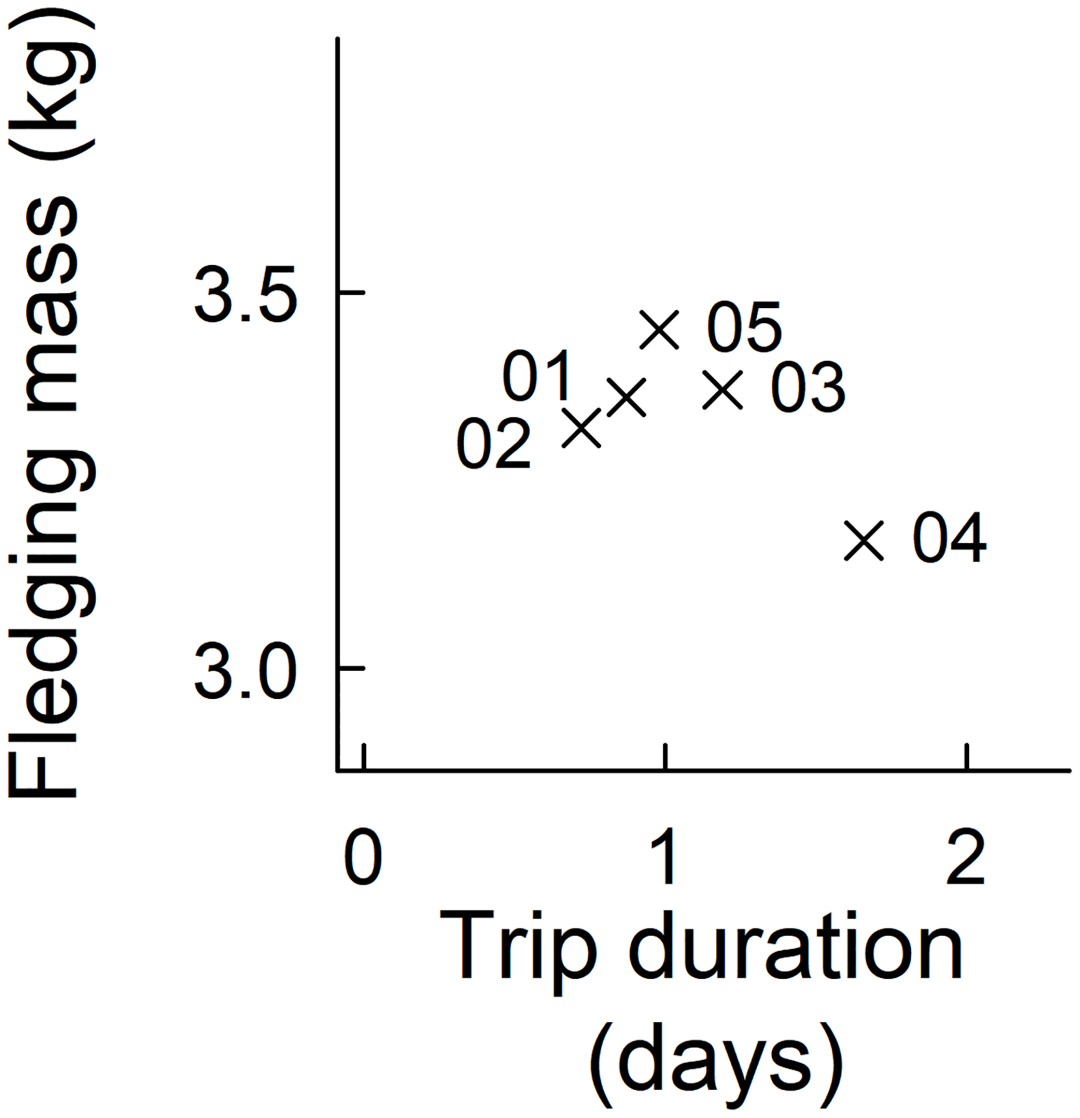
The relationship between macaroni penguin fledging mass and foraging trip duration during brood-guard, 2001–2005.

Fledgling mass differed between years, and chicks were significantly lighter during 2004 compared to all other years (ANOVA: F_4,511_ = 10.26, p<0.001, Fig 2C). Chicks also fledged with significantly heavier body masses during 2005, compared to 2002. Fledging mass was not significantly related to dive depth (lm: R^2^ = 0.76, F_2,2_ = 7.2, t = 2.59, p = 0.12), although the influence of trip duration was close to the 5% significance level (lm: t = -3.78, p = 0.06; Fig 4).

## Discussion

The activity budgets of macaroni penguins during early chick rearing were altered during 2004 when krill levels were very low. In this year, foraging trips were significantly longer, with most individuals travelling farther from the colony past the edge of the continental shelf. In addition, fledging masses were significantly lighter during 2004. Dietary analyses report the proportion of macaroni penguins with a diet dominated by krill was also much lower during 2004, compared to the other study years (2004 <10%, other years ~30–95%;[7]). A reduction in food supply is therefore the most obvious explanation for the observed changes in foraging behaviour and breeding investment.

Acoustic monitoring indicates that the biomass of krill at South Georgia is concentrated at the shelf edge [37]. In addition to 2004, individuals travelled to the shelf edge during 2003 and 2005, albeit with less prevalence. The shallower dive profiles observed during these three years may reflect the vertical distribution of krill in upwelling shelf-break waters [37]. Krill densities at South Georgia were reported as being much higher during 2003 and 2005, compared to 2004, however swarms were irregularly distributed [24]. Therefore, the observed foraging characteristics during these years may indicate that birds were unable to locate sufficient prey resources within the continental shelf zone.

In addition to 2004, local krill density was also very low during 2001. However, the coefficient of variance was higher in 2001, indicating more spatial variation in densities. In contrast to 2004, foraging was concentrated around the colony during 2001. In addition, trip durations did not significantly increase, although they were slightly longer than observed during 2002. Both metrics of diving behaviour were highly variable in 2001, and the annual mean for distance travelled at the bottom of a dive was the lowest observed during the study. Studies of diving behaviour in seals report that individuals are more likely to end dives earlier when no prey is found, compared to when a single prey item is encountered early in the dive [38,39]. However, the proportion of individuals with a diet dominated by krill was greater than 90% in 2001 [7]. Furthermore, fledging masses were not adversely impacted. Consequently, it appears that during 2001 birds were able to access sufficient inshore aggregations of krill that were possibly distributed in smaller, less dense swarms.

Maximum dive depth and distance travelled during the bottom phase of a dive were both elevated during 2002. Trips were also slightly shorter and birds were predominantly foraging closer to the colony. The density of krill at South Georgia was high with low spatial variability during 2002 (137 g m^-2^, CV = 30 [24]), however the proportion of macaroni penguins with a diet dominated by krill was lower than observed in 2001 and 2005 (2002 ~ 40%, 2001 < 90%, 2005 ~ 60%; [7]). A study using stable isotope analysis to examine intrapopulation variation in resource use also reported greater variation in the dietary signature of macaroni penguins during 2002 [40]. The distribution of krill within the water column can vary spatially and interannually depending on climate, prevailing currents and ocean temperature [37,41]. Diet information and diving behaviour for 2002 may therefore indicate that large swarms of krill did occur close to the colony but in deeper water such that accessibility to foraging macaroni penguins was reduced, and an alternative prey resource was targeted.

Macaroni penguins predominantly foraged directly north of the colony overlapping the area surveyed for krill density. Low levels of foraging activity were also observed directly south of the colony during 2005. This overall distribution is in agreement with GPS data collected from the same population during the brood-guard phase of an independent year [42]. Individual consistencies in trip distance and duration were also identified based on the mixed effect models. Evidence for specialisation in foraging distance, possibly associated with body size, has been previously reported for this population during the long-range foraging trip conducted prior to moult [42]. Future studies using modern telemetry devices would be able to conduct longer-term deployments with greater sample sizes to elucidate levels of individual variation further. Consistencies in foraging behaviour during brood-guard have been linked to age in little penguins(*Eudyptula minor*; [43]), and recent demography studies of this population [44] provide a basis for examining the influence of individual foraging performance as a function of age or experience.

Meal masses (g) and meal energy (kJ) delivered to macaroni penguin chicks did not change during 2004 when levels of krill density were low at South Georgia [7]. Therefore, extended trip durations (beyond 24hours) by breeding adults will have certainly reduced the daily rate of chick provisioning during this year (kJ day^-1^). A decrease in chick growth rates and fledging mass in response to adverse prey conditions has been previously reported for several penguin species [6,23,45–48], and our study indicates that altered activity budgets are a possible behavioural mechanism linking perturbations in prey availability to provisioning rates and fledging masses. Survival rates of macaroni penguins during the fledging year are also influenced, at least partially, by their fledging mass, and both fledging masses and survival rates were lower for the 2004 cohort [44]. Sub-adult life stages can play a key role in shaping the population dynamics of penguins [49–52], consequently the proposed linkages could contribute towards the population response of macaroni penguins to future climate or anthropogenic changes in krill abundance.

## Conclusions

Macaroni penguins extended their foraging trips overnight when krill densities were very low, possibly in order to locate sufficient resources in more predictable foraging grounds with lower levels of competition. This strategy resulted in lower fledging masses due to reduced rates of provisioning, and is also likely to have contributed towards lower first-year survival rates in that cohort. Our results highlight a possible behavioural mechanism that links perturbations in prey availability to population demography.

## Supporting information

S1 Table Times used to determine day from night activity. (DOCX)
