# Supplementary material for: Linking extreme interannual changes in prey availability to foraging behaviour and breeding investment in a marine predator, the macaroni penguin
Source: PLoS One. 2017 Sep 14;12(9):e0184114. doi: 10.1371/journal.pone.0184114 (PMC5598940; doi:10.1371/journal.pone.0184114)
Supplement: S1 Table — (DOCX) [file pone.0184114.s001.docx]

## **Supplementary material for**

## **Horswill et al*.*** Linking extreme inter-annual changes in prey availability to foraging behaviour and breeding investment in a marine predator, the macaroni penguin

Table S1; Times used to determine day from night activity. The distinction between local day and night for South Georgia was defined using nautical twilight incorporating global location, date and GMT offset (<http://www.sunrisesunset.com/custom_srss_calendar2.asp>; accessed 10 June 2011).

| Year | Day starts | Night starts | Duration of night |
| --- | --- | --- | --- |
| 2001 | 02:03:00 | 23:06:00 | 02:57:00 |
| 2002 | 01:53:30 | 23:26:30 | 02:27:00 |
| 2003 | 01:45:00 | 23:17:30 | 02:27:30 |
| 2004 | 01:44:00 | 23:34:00 | 02:10:00 |
| 2005 | 01:40:30 | 23:20:00 | 02:20:30 |
